# NEDD4L-induced ubiquitination mediating UBE2T degradation inhibits progression of lung adenocarcinoma via PI3K-AKT signaling

**DOI:** 10.1186/s12935-021-02341-9

**Published:** 2021-11-27

**Authors:** Yongbing Chen, Haihua Hong, Qingqing Wang, Junqiang Li, Wenfeng Zhang, Tingting Chen, Pu Li

**Affiliations:** 1 Department of Respiratory Medicine, Beilun Branch, Zhejiang University School of Medicine First Affiliated Hospital, Ningbo, 315800 China; 2 Department of Pathology, Beilun Branch, Zhejiang University School of Medicine First Affiliated Hospital, Ningbo, 315800 China; 3 Department of Infectious Disease, The First Affiliated Hospital, Nanchang University, Nanchang, 330052 China; 4 Department of Urology, The Second Affiliated Hospital, Third Military Medical University (Army Medical University), Chongqing, 400037 China; 5 State Drug Clinical Trial Agency, The First Affiliated Hospital, Nanchang University, Nanchang, 330052 China

**Keywords:** Lung adenocarcinoma, NEDD4L, UBE2T, Ubiquitylation, PI3K-AKT signaling

## Abstract

**Background:**

A number of studies have indicated that Ubiquitin-conjugating enzyme E2T (UBE2T), as an oncogene, promotes progression and metastasis of lung cancer, including lung adenocarcinoma (LUAD), but it is completely unknown whether and how UBE2T is ubiquitylated and degraded, and by which E3 ligase. NEDD4L plays a critical role in the regulation of cellular processes of various cancers, most of which is attributed to its E3 ubiquitin ligase function. However, the relationship between NEDD4L and UBE2T in LUAD has not been elucidated.

**Methods:**

The relationship between NEDD4L and UBE2T in LUAD tissues and cells was found by bioinformatic analyses and immunoblotting. Cell counting kit-8, colony formation assay, half-life analysis and the in vivo ubiquitylation assay, generation of xenograft model were performed to determine how NEDD4L regulates UBE2T and its downstream signaling pathway in vitro and in vivo.

**Results:**

Bioinformatic analyses found that NEDD4L, as a potential correlation E3 ligase of UBE2T, was negatively correlated with UBE2T in LUAD. Consistently, UBE2T protein half-life was shortened or extended by NEDD4L overexpression or depletion, respectively. NEDD4L inhibited LUAD cell progression in vitro and in vivo via inducing the ubiquitination-mediated UBE2T degradation, which repressed PI3K-AKT signaling. Similarly, NEDD4L predicted a better patient survival, whereas UBE2T predicted a worse survival.

**Conclusions:**

Collectively, our results reveal that NEDD4L is a novel E3 ligase of UBE2T, which can inhibit PI3K-AKT signaling by targeting for UBE2T ubiquitination and degradation, resulting in repression of LUAD cell progression.

## Introduction

Lung cancer is still the leading cause of cancer-related death around the world [1], and late diagnosis is a fundamental obstacle to improving lung cancer methods [2]. Histologically, most lung cancers (80%) are classified as non-small cell lung cancer (NSCLC), in which lung adenocarcinoma (LUAD) is the most prevalent subtype [3]. Importantly, accumulating studies have indicated that dysregulation of tumor related gene is commonly found in lung cancer [4], including mutations of the tumor suppressor gene p53 and activation of proto-oncogene K-Ras [5, 6]. Recently, the dysregulation of various components of the ubiquitin–proteasome system (UPS), which maintains protein homeostasis by degrading unwanted proteins in time [7–9], has been confirmed to be related to lung cancer [10–12].

The UPS can degrade proteins that affects many physiological functions in humans in a three-step enzyme cascade [8]. The ubiquitination process involves E1 ubiquitin-activating enzyme, E2 ubiquitin-conjugating enzyme and E3 ubiquitin ligase. Previous studies have shown that E2 enzyme ubiquitin-binding enzyme E2T (UBE2T) plays a carcinogenic role in most tumors by promoting the targeted degradation of multiple tumor suppressors, including FOXO1 [13], p53 [14], BRCA1 [15] and among many others. In vitro and in vivo studies showed UBE2T acted as an oncogene whose overexpression promoted cell progression and metastasis [13, 16], whereas its silencing by siRNA or deletion by gene knockout losed that function [17–19]. On the one hand, UBE2T could promote gastric cancer cell progression via hyperactivating the Wnt/β-catenin pathway through the ubiquitination and degradation of RACK1 [16]. On the other hand, UBE2T also could mediate H2AX/γH2AX monoubiquitylation on facilitating cell cycle arrest activation to provide sufficient time for radiation-induced DNA repair, thus conferring hepatocellular carcinoma radio-resistance [20]. Consistently, overexpressed UBE2T was associated with poor prognosis in gastric cancer [16], hepatocellular carcinoma [20], osteosarcoma [17] and breast cancer [15]. Recently, several lines of investigation revealed that UBE2T promoted proliferation and EMT by ubiquitination-mediated FOXO1 degradation and Wnt/β-catenin signaling pathway activation in NSCLC cells [13, 18]. So far, it is unknown how UBE2T is regulated at the post-translational level and by which E3 ligase.

Neural precursor cell expressed, developmentally down-regulated 4, E3 ubiquitin protein ligase (NEDD4) is a member of E3 ubiquitin ligase family with HECT domain. It is divided into two subtypes: NEDD4 (NEDD4-1) and NEDD4L (NEDD4-2) [21]. Although NEDD4 and NEDD4-L are commonly expressed, the two proteins have different functions by targeting specific proteins for ubiquitination [22]. NEDD4L has been reported to promote the degradation of certain proteins involved in tumor signaling pathways, including Dvl2 and Smad2/Smad3. Degradation of Dvl2 led to inhibition of the Wnt signaling [23], while degradation of Smad2/Smad3 resulted in the down-regulation of TGF-β signaling [24]. Both are closely related to the regulation of tumor progression. In lung cancer, NEDD4L was correlated negatively with cell survival and metastasis [25, 26]. But it also promoted lung cell survival by targeting GCN2 degradation [27]. Therefore, the mechanism of NEDD4L in lung cancer remains to be further elucidated.

In our current study, we outlined the expression and function of NEDD4L and UBE2T, hoping to gain insight into their roles in LUAD. Herein, we reported that NEDD4L had tumor suppressor activity to be against LUAD cells and blocked oncogenic function of UBE2T. Mechanistically, NEDD4L inhibited PI3K-AKT signaling via targeting for UBE2T ubiquitination and degradation, resulting in repression of LUAD cell progression. According to our results, UBE2T was added to a growing list of NEDD4L substrates, and NEDD4L/UBE2T played a crucial role in LUAD cell progression.

## Materials and methods

### Reagents and cell cultures

MG-132(#HY-13259, MCE) and Cycloheximide (#HY-12320, MCE) were purchased from MedChemExpress, and dissolved in dimethyl sulfoxide (DMSO) (#N182, Amresco) and stored at − 20 °C. Immortalized human bronchial epithelial cell line (Beas-2B) was obtained from Dr. Jie Xu from Third Military Medical University and cultured in BEBM medium. The human LUAD cell lines (H1299, H358, A549, H23 and H1975) were available from the Zhong Qiao Xin Zhou Biotechnology Co., Ltd. (Shanghai, China). H1299, H358, H23 and H1975 were cultured in RPMI-1640 medium with 10% fetal bovine serum (FBS) (#35-081-CV, Corning) and 1% Penicillin/ Streptomycin (#15140122, Gibco). A549 was grown in F-12K medium (#11765054, Gibco) with 10% FBS. All the medium was purchased from Gibco. All cell lines were tested to be free of mycoplasma contamination.

### Cell proliferation assay

Cell proliferation was determined by the Cell Counting Kit-8 (CCK-8) assay (#HY-K0301, MCE), according to the manufacturer’s instructions. The transfected H1299 and H358 cells concentration was adjusted to 2 × 10^3^ cells/well, and the cells were seeded into 96-well plates in triplicate. At different time after cell plating, the viability of cells was determined by measuring the optical density (OD) at 450 nm.

### Immunoblotting and immunoprecipitation

Cells were harvested, lysed and subjected to direct immunoblotting (IB) or immunoprecipitation (IP) as previously described [28]. Briefly, cells were lysed in cell lysis buffer (20 mM Tris–HCL pH 7.5, 150 mM NaCl, 1% NP-40, 0.1% SDS, 0.5% sodium deoxycholate, 1 mM EDTA, 1 mM Na_3_VO_4_, 50 mM NaF) with protease inhibitors (#11873580001, Roche) and phosphatase inhibitors (#04906837001, Roche), and incubated on ice for 30 min. The supernatants were harvested by spinning at 12,000 rpm for 20 min at 4 °C. The same amounts of whole cell lysates were subjected to IB after the protein concentration measured using the BCA protein assay kit (#P0009 Beyotime Biotechnology). To immunoprecipitate exogenously expressed HA-tagged proteins or endogenous proteins, the supernatants were incubated with bead-conjugated with HA antibody (1:1000, #PA1-29751, Cell Signaling Technology) or according antibodies followed by Protein A/G PLUS-Agarose beads (#sc-2003, Santa Cruz) in a rotating incubator for 4 h at 4 °C. The immunoprecipitates were washed with cell lysis buffer for three times and then subjected to IB. The primary antibodies used for IB were as follows: UBE2T (1:1000, #12992), NEDD4L (1:1000, #4013), Cleaved Caspase-3 (Asp175) (5A1E) (1:1000, #9664), p21 (1:1000, #2947), AKT (pan) (40D4) (1:1000, #2920) and Phospho-AKT (Ser473) (D9E) XP (1:1000, #4060) were purchased from Cell Signaling Technology. GAPDH (1:1000, #60004-1-Ig) was purchased from Proteintech. FLAG was obtained from Sigma-Aldrich (#F1804). As well as all secondary antibodies were purchased from Cell Signaling Technology (Danvers, MA, USA).

### Plasmids, siRNA, and transfection

Plasmids of HA-NEDD4L and FLAG-UBE2T were obtained from Dr. Jie Xu from Third Military Medical University. The siRNA targeting NEDD4L (#sc-75894) and UBE2T (#sc-78641) are all pools purchased from Santa Cruz. Each pool contains 3–5 target-specific 19–25 nt siRNAs. The sequence for the scrambled control siRNA is 5′-AUUGUAUGCGAUCGCAGACUU-3′ [29]. For transient transfection, cells were seeded in antibiotic-free medium at 37 °C for 24 h and transfected with targeting plasmids or siRNA using lipofectamine 2000 transfection reagent (#11668019, Invitrogen) in accordance with the manufacturer's instructions, and treated 48 h after transfection.

### Colony formation assay

Briefly, the transfected H1299 and H358 cells were seeded into 60-mm dishes (Corning, NY, USA) in triplicate at a density of 2000 cells/well, followed by incubation at 37 °C for 14 days. The colonies were fixed with 4% paraformaldehyde for 15 min, stained with crystal violet at room temperature for 30 min, and then counted [30].

### Cycloheximide chase analysis

Cycloheximide chase analysis was performed to define the effect of NEDD4L on the stability of UBE2T protein as described previously [28]. After transfection with relevant plasmids for 48 h, 293 cells were switched to fresh medium (10% FBS) containing 50 µg/mL cycloheximide (CHX), followed by collection of transfected cells at indicted time points for IB assay. Each experiment was conducted in triplicate.

### Quantitative real-time reverse transcription PCR (qRT-PCR)

Total RNA was extracted from cells by RNAisoPlus (Takara, #9108), and reverse transcribed to cDNA with PrimeScript RT reagent Kit with gDNA Eraser (Takara Bio, Inc., Otsu, Japan), according to the manufacturer’s protocols. qRT-PCR was carried out using QuantiNova™ SYBR^®^ Green PCR Kit (Qiagen GmbH, Hilden, Germany) on an Applied Biosystems 7900HT Real-Time PCR System. GAPDH was used as the housekeeping gene. The following forward primers, and reverse primers were used: GAPDH forward: 5′-ATCACCATCTTCCAGGAGCGAG-3′, GAPDH reverse: 5′-GGGCAGAGATGATGACCCTTTTG-3′; UBE2T forward: 5′-CAAATATTAGGTGGAGCCAACAC-3′, UBE2T reverse: 5′-TAGATCACCTTGGCAAAGAACC-3′. The mRNA relative expression levels of UBE2T were calculated by 2^−ΔΔCt^ quantification method. Each experiment was conducted in triplicate.

### The in vivo ubiquitylation

The 293 cells were co-transfected with FLAG-UBE2T, HA-NEDD4L and His-Ub, along with vector control. In vivo ubiquitylation assays were performed as previously described using Ni-beads pull-down [12]. And cells were treated with MG132 (10 μM) for another 4 h before lysed.

### In vivo xenograft model

All animal-related experiments were carried out according to our protocol approved by the University Committee for Use and Care of Animals. Four- to six-week-old BALB/c athymic nude mice (nu/nu, female) were used with each experimental group consisting of four mice. H1299 cells were seeded in antibiotic-free medium at 37 °C for 24 h and transfected with FLAG-UBE2T plasmid using lipofectamine-2000 transfection reagent according to the manufacturer’s instructions, and treated 48 h after transfection. Then, the obtained stable cells were transfected with HA-NEDD4L plasmid using lipofectamine-2000 transfection reagent, and treated 48 h after transfection. In total, approximately 1 × 10^6^ transfected H1299 cells (Vector, FLAG − UBE2T, FLAG − UBE2T + HA − NEDD4L) were mixed 1:1 with matrigel (#354230, BD biosciences) in a total volume of 0.2 mL and were injected subcutaneously into right flank of mice (n = 4 per group). The body weights and growth of tumor were measured twice a week. The tumor volume (TV) was calculated according to the equation: TV = (L × W^2^)/2, where L is the length and W is the width of the tumor [28]. After four weeks, the mice were placed in a clean cage. CO_2_ is then injected into the cage at a rate of 2L/min. When the mice stopped breathing (about 5 min), cervical dislocation was performed. Tumors were collected for paraffinembedding and immunohistochemical staining. All animal experiments were reviewed and approved by the Institutional Animal Care and Use Committee of Model Animal Research Center of Army Medical University (Third Military Medical University) of China.

### Clinical specimens

A total of 4 LUAD tissue samples and matched non-tumor adjacent tissues specimens were derived from biopsy samples and were finally confirmed. Detailed Clinicopathological data of the patients is shown in Table 1. The study was conducted in accordance with the Declaration of Helsinki, and the protocol was approved by People’s Hospital of Beilun District, Ningbo, China. All patients signed the informed consent.

**Table 1:** Clinicopathological data of the patients

ID	Gender	Age	Pathologic types	TNM stage
2291078	Female	52	Infiltrating adenocarcinoma of the right upper lung	T_1c_N_0_M_0_
2291875	Male	57	Infiltrating adenocarcinoma of the right middle lung	T_1b_N_0_M_0_
2295361	Male	65	Infiltrating adenocarcinoma of the lower left lung	T_2a_N_0_M_0_
3397749	Male	51	Infiltrating adenocarcinoma of the left upper lung	T_1c_N_0_M_0_

### Immunohistochemical staining

Immunohistochemical (IHC) staining of mice tumors was performed as described previously [31]. Briefly, after deparaffinization, rehydration, antigen retrieval and blocking, the tissue slides were incubated overnight at 4 °C with indicated antibodies. The following primary antibodies were used: Ki-67 (1:1000, #9449, Cell Signaling Technology), p21 (1:200, 10355-1-AP, Proteintech) and Cleaved-capase-3 (Asp175) (5A1E) (1:200, #9664, Cell Signaling Technology).

### Statistical analysis

SPSS statistical software (version 22.0) was used for statistical analysis of the experimental data. Welch’s test was conducted for differential expression of functional mRNA in UALCAN. Transcripts per million (TPM) values and Student’s t tests were employed to calculate the significance of gene expression divergence between categories in GEPIA. Log-rank test was implemented in GEPIA for comparison of overall survival curves, which displayed as Kaplan–Meier plot. All data were shown as the average ± standard deviation (Mean ± SD). *P* < 0.05 is considered significant in this entry (*P < 0.05; **P < 0.01; ***P < 0.001).

## Results

### Integrated analysis of lung cancer reveals that NEDD4L may be an E3 ligase for UBE2T

**Fig. 1:**
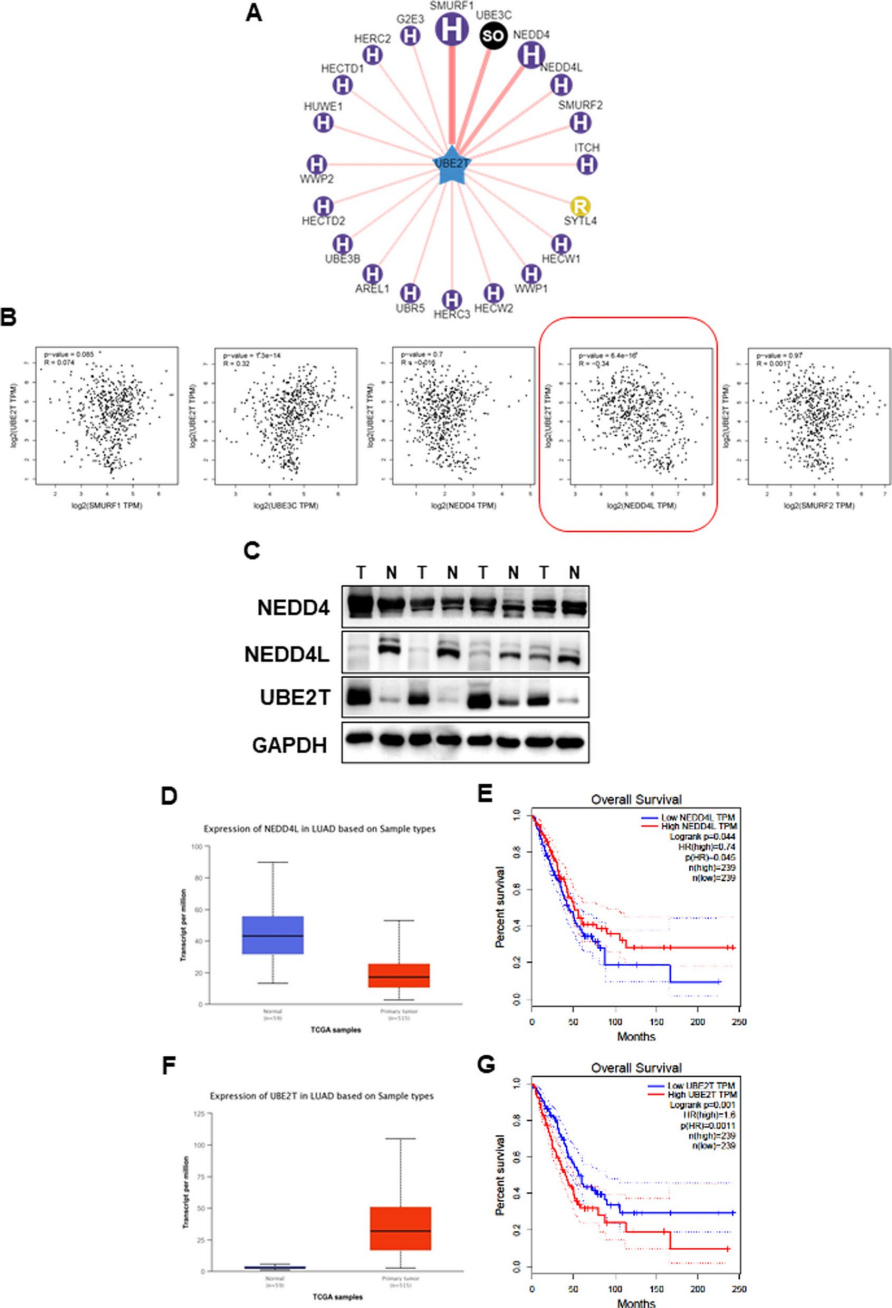
NEDD4L may be an E3 ligase for UBE2T. **A** Bioinformatics analysis came from http://ubibrowser.ncpsb.org.cn/ubibrowser/. **B** Among top five E3 ligase proteins, NEDD4L was extremely negative correlation with UBE2T, and data from http://gepia.cancer-pku.cn/. **C** NEDD4 family and UBE2T protein levels in LUAD tissues and adjacent normal tissues were analyzed by immunoblotting (IB) analysis. **D**, **F** Bar graph showing expression values of the identified genes using the study dataset, and data came from http://ualcan.path.uab.edu. The 95% confidence interval was also displayed. **E**, **G** Protein expression of NEDD4L and UBE2T in LUAD and their relationship with patient survival: Kaplan–Meier survival analysis indicated that patient with higher expression of NEDD4L was related to a better overall survival (log-rank test, *P* = 0.045) (**E**); While higher expression of UBE2T was related to a worse overall patient survival (log-rank test, *P* = 0.0011) (**G**)

UBE2T was highly expressed in lung cancer, and its protein expression was negatively correlated with the survival of lung cancer patients [13, 17]. However, how UBE2T is ubiquitinated and degraded, and which protein its E3 ligase is, remains unclear. To further elucidate that, a genetic bioinformatics database, UbiBrowser, was searched and found 20 potential correlation E3 ligase proteins of UBE2T (Fig. 1A). Among top five E3 ligase proteins, NEDD4L was extremely negative correlation with UBE2T in mRNA level (Fig. 1B). We next examined protein expression of NEDD4 family members (NEDD4 and NEDD4L) and UBE2T by immunoblotting (IB) in LUAD and adjacent normal tissues (Fig. 1C). UBE2T was highly expressed in LUAD tumor tissues, in contrast, NEDD4L was lowly expressed. Expression of NEDD4 had no difference in neither LUAD tumor tissues nor adjacent normal tissues. And as well as the down-regulation of NEDD4L and the up-regulation of UBE2T in LUAD were independently confirmed using the cancer genome atlas (TCGA) database (Fig. 1D, F). Kaplan–Meier survival analysis indicated that patients with higher expression of NEDD4L were related to a better overall survival (Fig. 1g), whereas patients with higher expression of UBE2T were related to a worse overall survival (Fig. 1E). According to integrated analysis, we hypothesized that E3 ligase NEDD4L as a potential binding partner of UBE2T in LUAD.

### NEDD4L binds to UBE2T and regulates UBE2T protein level

**Fig. 2:**
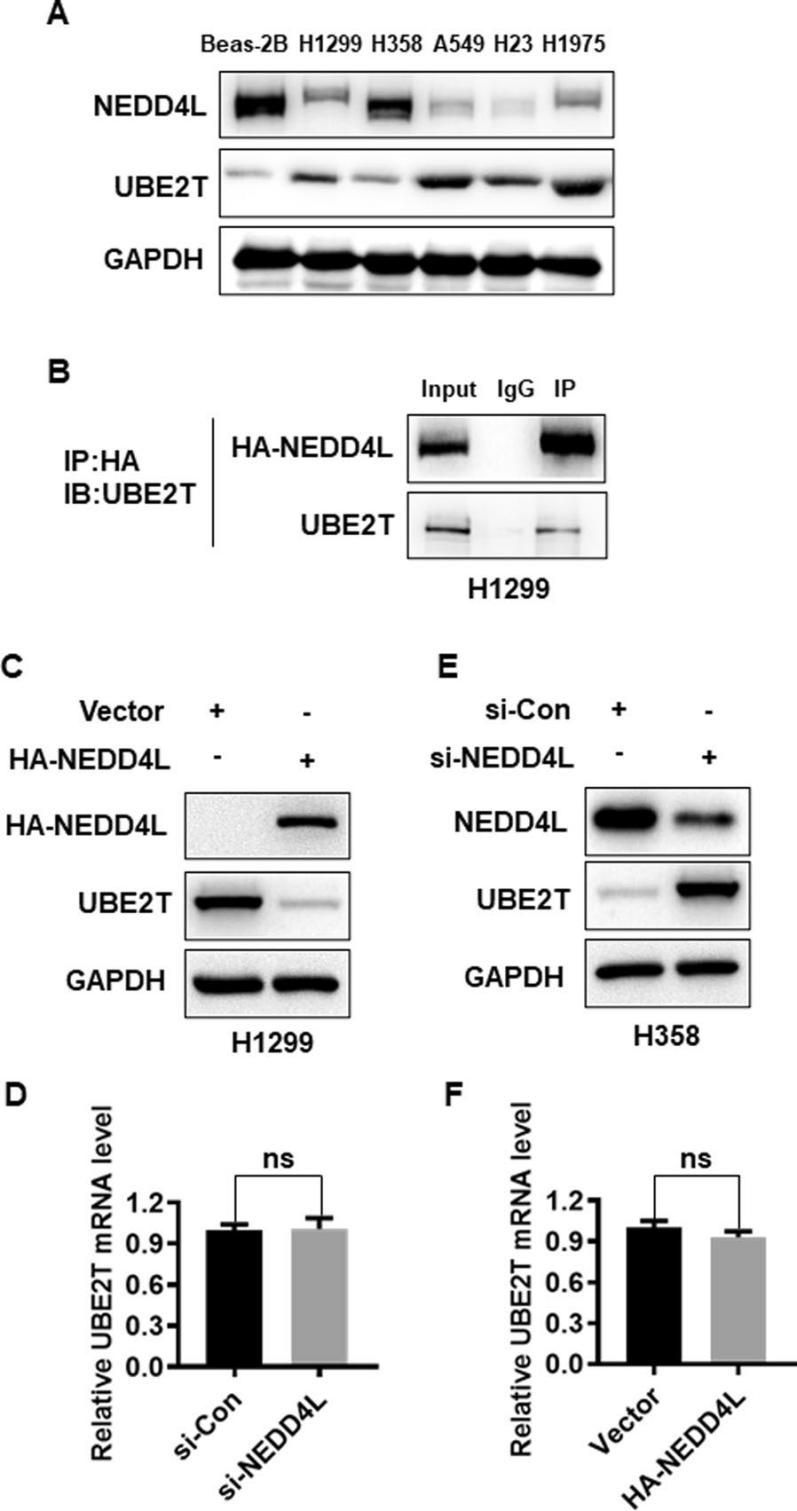
NEDD4L binds to UBE2T and regulates UBE2T levels. **A** Basal level of NEDD4L and UBE2T protein in lung cell lines. **B** NEDD4L immunoprecipitated endogenous UBE2T. H1299 cells were transiently transfected with HA-NEDD4L, then cells were lysed and immunoprecipitated with HA antibody, followed by IB with indicated antibodies. **C**, **D** Overexpression of NEDD4L decreased the protein level of UBE2T, but had no effect on UBE2T mRNA. H1299 cells were transfected with HA-NEDD4L or vector control, followed by IB **(C)** or qRT-PCR **(D)**. **E**, **F** NEDD4L depletion increased UBE2T protein level, but not mRNA: H358 cells were transfected with siRNA targeting NEDD4L along with control RNAi, followed by IB (**E**) or qRT-PCR (**F**)

We further investigated the relationship between the two proteins in LUAD cells, we measured their expression status by IB. Consistently, NEDD4L with highly expression and UBE2T with lowly expression were detected in LUAD cells, except H358 cells (Fig. 2A). Subsequent immunoprecipitation assays showed that UBE2T-NEDD4L binding was detected under ectopic overexpressed conditions (Fig. 2B). These results established an in vivo interaction between NEDD4L and UBE2T. Having detected a physical interaction between two proteins, we next determined whether UBE2T protein level is regulated by NEDD4L. We transfected HA-NEDD4L into H1299 cells, which showed a low protein level of NEDD4L and a moderate protein level of UBE2T (Fig. 2A), and found that the endogenous UBE2T protein level was significantly down-regulated (Fig. 2C). Furthermore, overexpression of NEDD4L had no effect on UBE2T mRNA level (Fig. 2D). Likewise, we performed an siRNA-based knockdown experiment in H358 LUAD cells with strong NEDD4L expression and weak UBE2T expression (Fig. 2A), and found endogenous levels of UBE2T protein, but not UBE2T mRNA, were increased upon NEDD4L knockdown (Fig. 2E, F). These data collectively indicated that NEDD4L could bind UBE2T and regulate UBE2T protein level, but not its mRNA.

### NEDD4L regulates UBE2T protein level by promoting its ubiquitylation

**Fig. 3:**
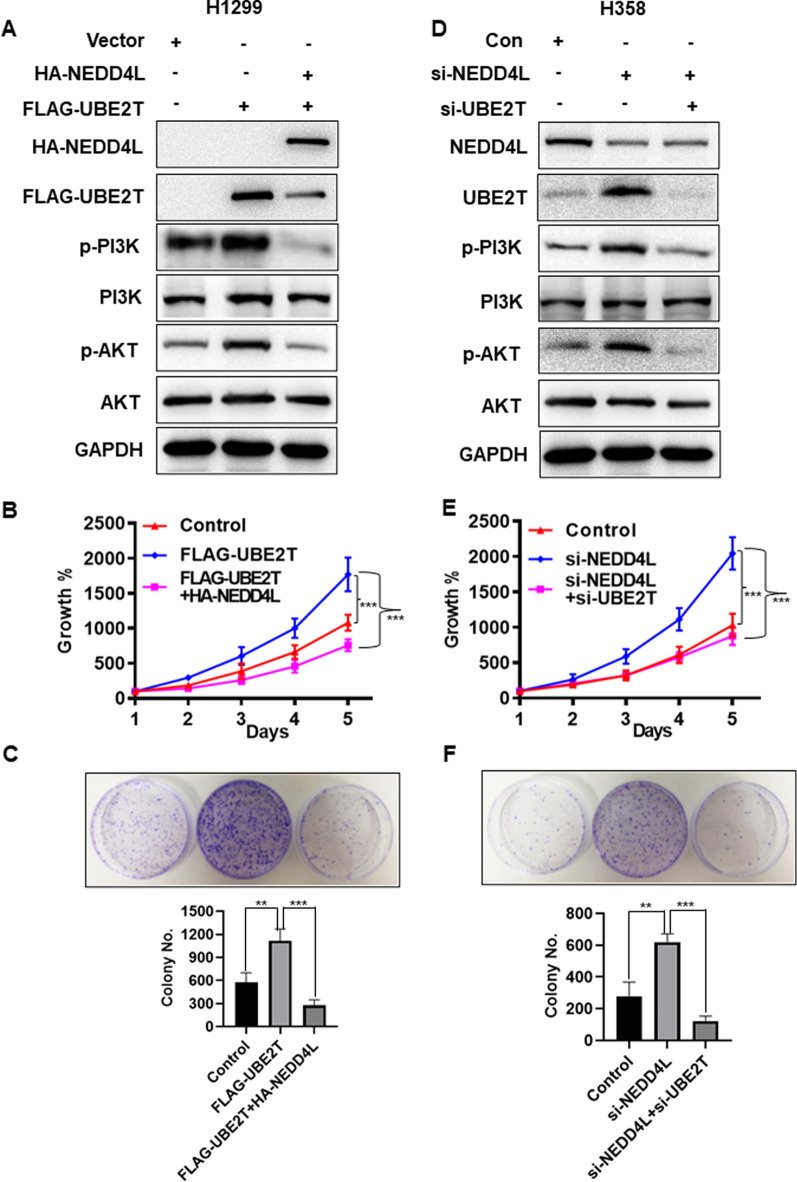
NEDD4L regulates UBE2T protein level by promoting its ubiquitylation. **A** Overexpression of NEDD4L decreased the protein level of UBE2T in a dose-dependent manner. H1299 cells were transfected with increasing amounts of HA-NEDD4L, followed by IB with indicated Abs. **B** Blockade of proteasome-mediated degradation rescued NEDD4L-mediating UBE2T down-regulation. H1299 cells were transfected with different doses of HA-NEDD4L (0, 0.5, 1 and 2 μg) for 48 h. The proteasome inhibitor, MG-132, was then added to cells and MG-132 co-treatment was allowed to occur for 8 h, followed by IB. **C** NEDD4L shortened the half-life of exogenous UBE2T protein. After transfection with relevant plasmids for 48 h, 293 cells were switched to fresh medium (10% FBS) containing cycloheximide (CHX) and incubated for indicated time periods before being harvested for IB. The band density was quantified using ImageJ software and plotted. **D** NEDD4L RNAi silencing extended protein half-life of endogenous UBE2T. H358 cells were transfected with either control RNAi, or Si-NEDD4L for 48 h. Cells were cultured in fresh medium containing CHX and incubated for indicated time periods before being harvested for IB. The band density was quantified using ImageJ software and plotted. **E** NEDD4L promoted UBE2T ubiquitylation in vivo: 293 cells were transfected with indicated plasmids, lysed under denatured condition at 6 M guanidinium solution, followed by Ni-beads pull-down. Washed beads were boiled for IB to detect polyubiquitylation of exogenous UBE2T

Having detected inversely correlated protein levels and a physical interaction between UBE2T and NEDD4L, we next determined whether UBE2T protein level is regulated by NEDD4L in dose- and time- manner. A dose-dependent reduction of endogenous UBE2T was detected when HA-NEDD4L was transfected (Fig. 3A). Moreover, NEDD4L-mediating UBE2T downregulation was significantly rescued when protein degradation was inhibited in the presence of MG132 (Fig. 3B). We then determined whether NEDD4L shortens the half-life of UBE2T. Indeed, while transfected FLAG-UBE2T remained stable after 16 h of cycloheximide (CHX) treatment, HA-NEDD4L co-transfection significantly reduced FLAG-UBE2T level and shortened its half-life (Fig. 3C). At the same time, NEDD4L depletion using siRNA in H358 cell line with expressing high levels of NEDD4L could lead to the accumulation of UBE2T protein, and the half-life of endogenous UBE2T protein was extended from 8 h to more than 24 h (Fig. 3D). To further determined whether UBE2T is a substrate of NEDD4L, in vivo ubiquitylation assay was performed to reveal it. The in vivo ubiquitylation assay showed that HA-NEDD4L significantly promoted ubiquitylation of FLAG-UBE2T in 293 cells (Fig. 3E). In summary, UBE2T was subject to NEDD4L-mediating ubiquitination and degradation. Therefore, we strongly supported the notion that UBE2T was a novel substrate for E3 ligase NEDD4L.

### NEDD4L mediates the biological effects of UBE2T

**Fig. 4:**
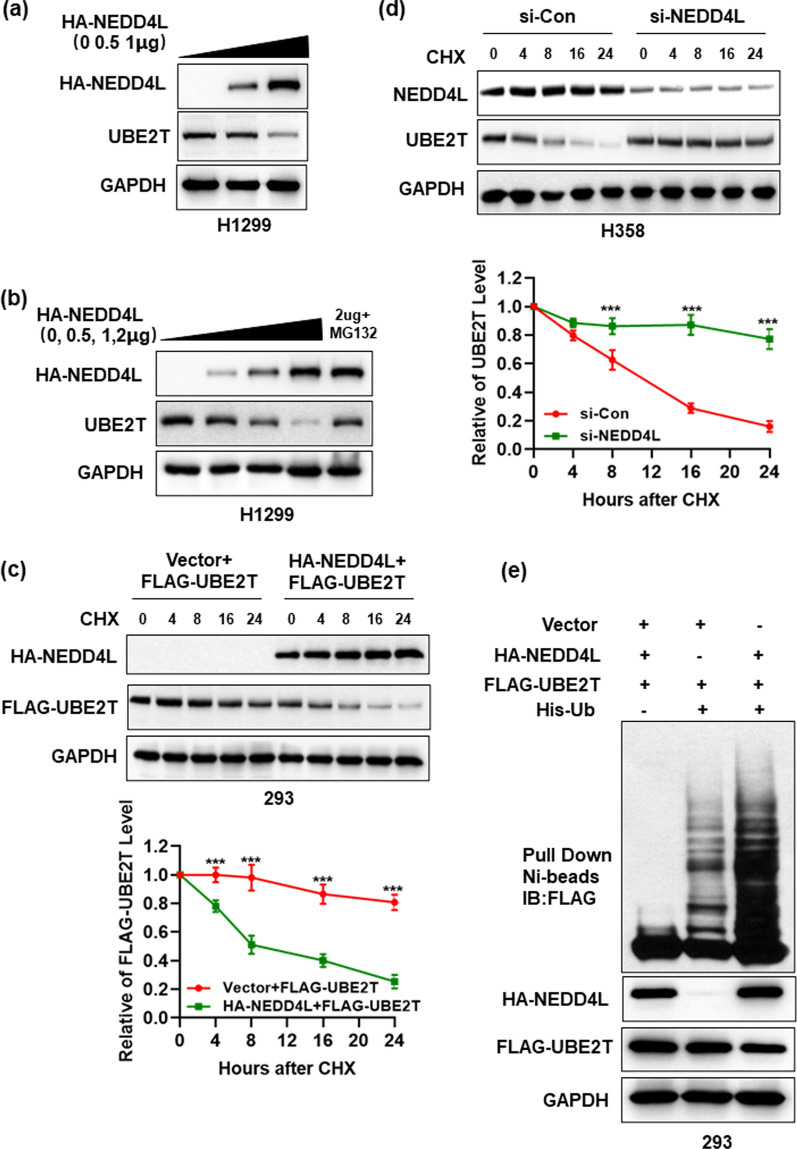
NEDD4L mediates the biological effects of UBE2T. **A** UBE2T overexpression increased level of p-PI3K and p-AKT, which was blocked by simultaneous transfection of HA-NEDD4L. H1299 cells were transfected with vector, FlAG-UBE2T, or FlAG-UBE2T in combination with HA-NEDD4L, followed by IB with indicated Abs. **B**, **C** NEDD4L arrested growth-promoting phenotype induced by UBE2T overexpression. H1299 cells were transfected with vector, FlAG-UBE2T, or FlAG-UBE2T in combination with HA-NEDD4L, followed by cell proliferation assay **(B)** and colony formation assay (**C**). **D** NEDD4L depletion caused UBE2T accumulation to increase level of p-PI3K and p-AKT, which was abrogated by simultaneous UBE2T depletion. H358 cells were transfected with si-NEDD4L, si-NEDD4L in combination with si-UBE2T along with control RNAi, followed by IB with indicated Abs. **E–F** NEDD4L depletion stimulated cell progression, which was abrogated by simultaneous UBE2T depletion: H358 cells were transfected with siRNAs targeting NEDD4L alone or in combination with siRNA targeting UBE2T, along with scramble control, followed by cell proliferation assay (**E**) and colony formation assay (**F**). Shown are mean ± SEM, **P < 0.01, ***P < 0.001

In Osteosarcoma and renal cell carcinoma, UBE2T overexpression increased the capacity of proliferation via PI3K/AKT pathway [17, 32], and was associated with aggressiveness, metastasis and poor prognosis [13, 18, 33, 34]. NEDD4L, on the other hand, is a tumor suppressor that targets various oncogenic proteins for degradation [23, 24]. We therefore confirmed whether NEDD4L targeting for UBE2T ubiquitination and degradation results in repression of PI3K/AKT signaling. Indeed, UBE2T overexpression increased level of p-PI3K and p-AKT, which could be blocked by simultaneous transfection of HA-NEDD4L (Fig. 4A). Conversely, NEDD4L depletion caused UBE2T accumulation to increase level of p-PI3K and p-AKT, which was abrogated by simultaneous UBE2T depletion both in vitro cell culture setting (Fig. 4D). We then performed biological rescue experiment to determine whether NEDD4L/UBE2T coordinately regulates LUAD cells progression. We transfected FLAG-UBE2T or FLAG-UBE2T in combination with HA-NEDD4L into H1299 LUAD cells, and found that transfection of FLAG-UBE2T alone stimulated the cell proliferation (Fig. 4B) and increased clonogenic survival of LUAD cells (Fig. 4C). This effect was blocked by simultaneous transfection of HA-NEDD4L. Furthermore, we transfected with si-NEDD4L, si-NEDD4L in combination with si-UBE2T along with control RNAi in H358 LUAD cells, and found that NEDD4L depletion caused UBE2T accumulation to stimulate cell proliferation (Fig. 4E) and clonogenic survival (Fig. 4F), which was abrogated by simultaneous UBE2T depletion both in vitro cell culture setting. Hence, reduction of UBE2T by siRNA silencing or overexpression of its E3 ligase NEDD4L could inhibit cell progression by regulating PI3K-AKT signaling, which further supported the view that NEDD4L/UBE2T played a crucial role in LUAD cell progression.

### NEDD4L mediates reduction of UBE2T in vivo

**Fig. 5:**
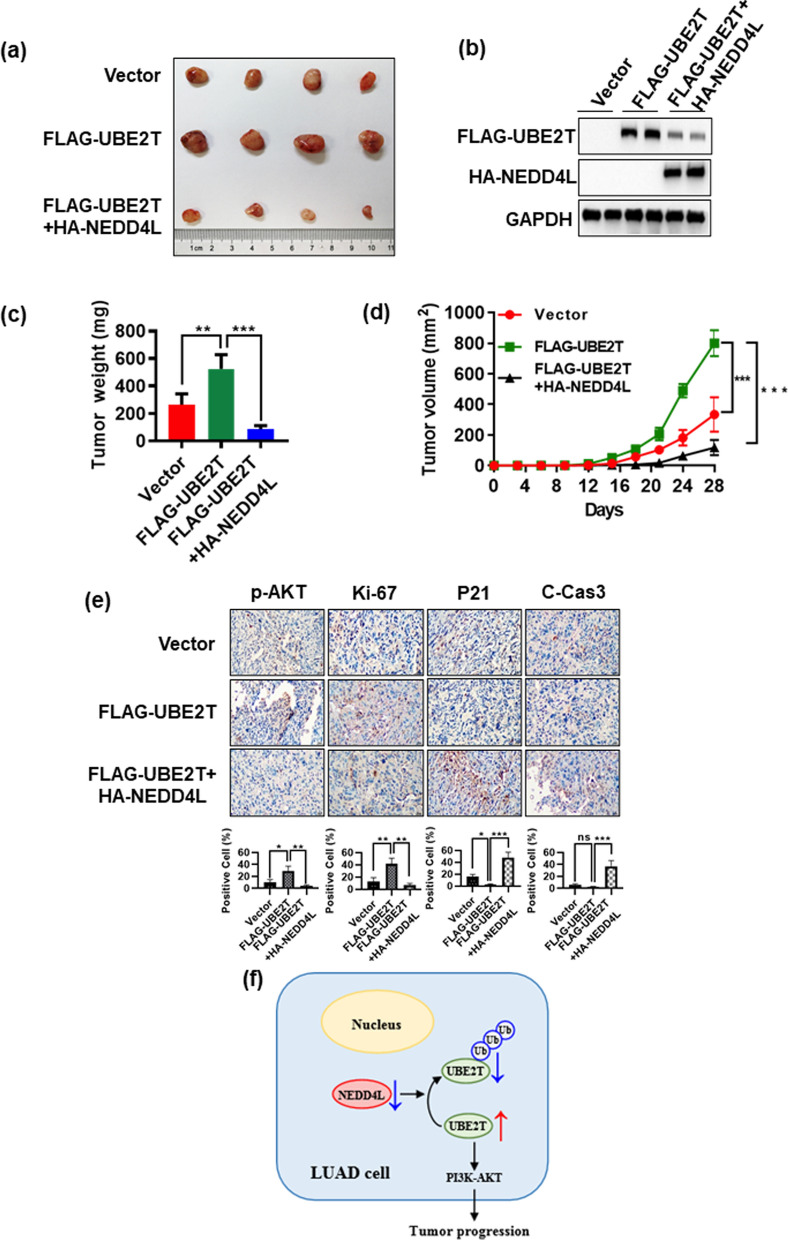
NEDD4L mediates reduction of UBE2T in vivo*.* Overexpression NEDD4L could result in UBE2T reduction to inhibit tumor growth in the H1299 xenograft model. The H1299 stable clones expressing vector, FLAG-UBE2T, or FLAG-UBE2T in combination with HA-NEDD4L into right flank side of nude mice. The tumors were harvested **(A)** and weighted **(C)**, and the tumor growth was monitored and growth curve was plotted (**D**). **B** UBE2T expression was reduced by NEDD4L in xenograft tumor tissues. The HA-NEDD4L and FLAG-UBE2T proteins were detected in xenograft tumor tissues by IB. **E** IHC staining of xenograft tumor tissues. Tumor tissues from three groups of mice were fixed, sectioned, and stained with indicated antibodies. Scale bars: 100 mm. **F** Schematic model for NEDD4L-induced UBE2T degradation inhibiting progression of LUAD cell. Shown are mean ± SEM, **P < 0.01, ***P < 0.001

We next determined the cross-talking of two proteins using an in vivo xenograft tumor model by inoculating subcutaneously the H1299 stable clones expressing vector, FLAG-UBE2T, or FLAG-UBE2T in combination with HA-NEDD4L into right flank side of nude mice. We found that increased tumor growth in vivo triggered by FLAG-UBE2T could be reversed by simultaneous transfection of HA-NEDD4L (Fig. 5A), due to overexpression of NEDD4L caused UBE2T reduction (Fig. 5B). And the average tumor size (Fig. 5A) and tumor weight (Fig. 5C) at the end of experiment (Day 28) were significantly lower in the combinational group. Noted that tumor growth rate was the lowest in combinational group (Fig. 5D), which was consistent with the in vitro results. Finally, IHC staining of tumor tissues revealed that UBE2T-expressing tumor had increased proliferation index (increased Ki-67 and decreased P21), reduced apoptosis index (C-Cas3) and increased p-AKT expression, which was abrogated by simultaneous transfection of HA-NEDD4L (Fig. 5E). Taken together, the results of in vitro cell culture and in vivo xenograft models consistently showed that NEDD4L-mediating UBE2T degradation could inhibit the progression of LUAD cells, and ultimately reduced tumor formation.

## Discussion

NEDD4L triggers the degradation of certain proteins involved in cancer progression, most of which is due to its E3 ubiquitin ligase function [16, 20, 35]. Meanwhile, UBE2T overexpression has also been found in a variety of cancers. Although most studies have reported that UBE2T and NEDD4L are involved in the growth regulation of lung cancer cells [13, 18, 27], their cross-role in the synergistic regulation of cancer cell growth has never been reported before. In this study, we identified UBE2T was negatively regulated by NEDD4L E3 ubiquitin ligase at the post-translational level. Our conclusion is supported by the following lines of evidence: (1) NEDD4L binds to UBE2T under the physiological conditions; (2) Cellular levels of UBE2T can be decreased or increased by NEDD4L overexpression or depletion, respectively; (3) The half-life of UBE2T protein is shortened or extended by NEDD4L overexpression or depletion, respectively; (4) NEDD4L leads to UBE2T ubiquitylation. NEDD4L has been shown bind to and ubiquitylate many cellular proteins for targeted degradation [35, 36], but has never about it targets UBE2T. Taken together, we first report that UBE2T is a novel physiological substrate of the E3 ubiquitin ligase NEDD4L.

Emerging evidences have reported that NEDD4L may function as a tumor suppressor that is frequently reduced in human carcinomas of pancreatic [37], colorectal [35], melanoma [36], ovarian [38] and lung [25, 26]. But some studies reported that NEDD4L promoted lung cancer cell survival [27]. Therefore, biological function of NEDD4L in lung cancer is still unclear. Here we showed that NEDD4L acted as a tumor suppressor in LUAD with following lines of supporting evidence. (1) Ectopic expression of UBE2T significantly promotes the growth and clonogenic survival of LUAD cells, which is similar to a previous study [13], but it can be reversed by NEDD4L overexpression; (2) siRNA-based depletion of NEDD4L promotes the growth and clonogenic survival of LUAD cells, which is abrogated by simultaneous UBE2T depletion; (3) NEDD4L is down-regulated in lung cancer tissues and high levels of NEDD4L predicts a better patient survival, whose results are consistent with previous studies [25, 26]. (4) NEDD4L targets ubiquitylation and degradation of UBE2T, which has been reported that induce increase of phosphorylation of PI3K and AKT [17, 32], therefore, tumor suppressor function of NEDD4L is mediated by suppression PI3K-AKT pathway. Collectively, functional studies have revealed that growth-inhibiting effect of NEDD4L is mediated by targeting ubiquitylation and degradation of UBE2T.

## Conclusions

In summary, NEDD4L is a novel E3 ligase for targeting ubiquitination and degradation of UBE2T, and resulting in repressing PI3K-AKT signaling, which inhibits LUAD progression (Fig. 5F). Thus, UBE2T is added to a growing list of NEDD4L substrates, and NEDD4L/UBE2T plays a crucial role in LUAD.

## Data Availability

The data that support the findings of this study are openly available in UbiBrowser (http://ubibrowser.ncpsb.org.cn/ubibrowser/), GEPIA (http://gepia.cancer-pku.cn/) and Kaplan–Meier survival analysis (http://ualcan.path.uab.edu). The rest of the data are available from the corresponding author on reasonable request.

## Abbreviations

LUAD: Lung adenocarcinoma
NSCLC: Non-small cell lung cancer
UPS: Ubiquitin–proteasome system
UBE2T: Ubiquitin-binding enzyme E2T
NEDD4: Neural precursor cell expressed, developmentally down-regulated 4, E3 ubiquitin protein ligase
FBS: Fetal bovine serum
CCK-8: Cell counting Kit-8
OD: Optical density
IB: Immunoblotting
IP: Immunoprecipitation
qRT-PCR: Quantitative real-time reverse transcription PCR
CHX: Cycloheximide
TV: Tumor volume
IHC: Immunohistochemical
TPM: Transcripts per million
TCGA: The cancer genome atlas
